# Supplementary figures and images for: Foraging niche overlap during chick-rearing in the sexually dimorphic Westland petrel
Source: R Soc Open Sci. 2020 Nov 25;7(11):191511. doi: 10.1098/rsos.191511 (PMC7735354; doi:10.1098/rsos.191511)

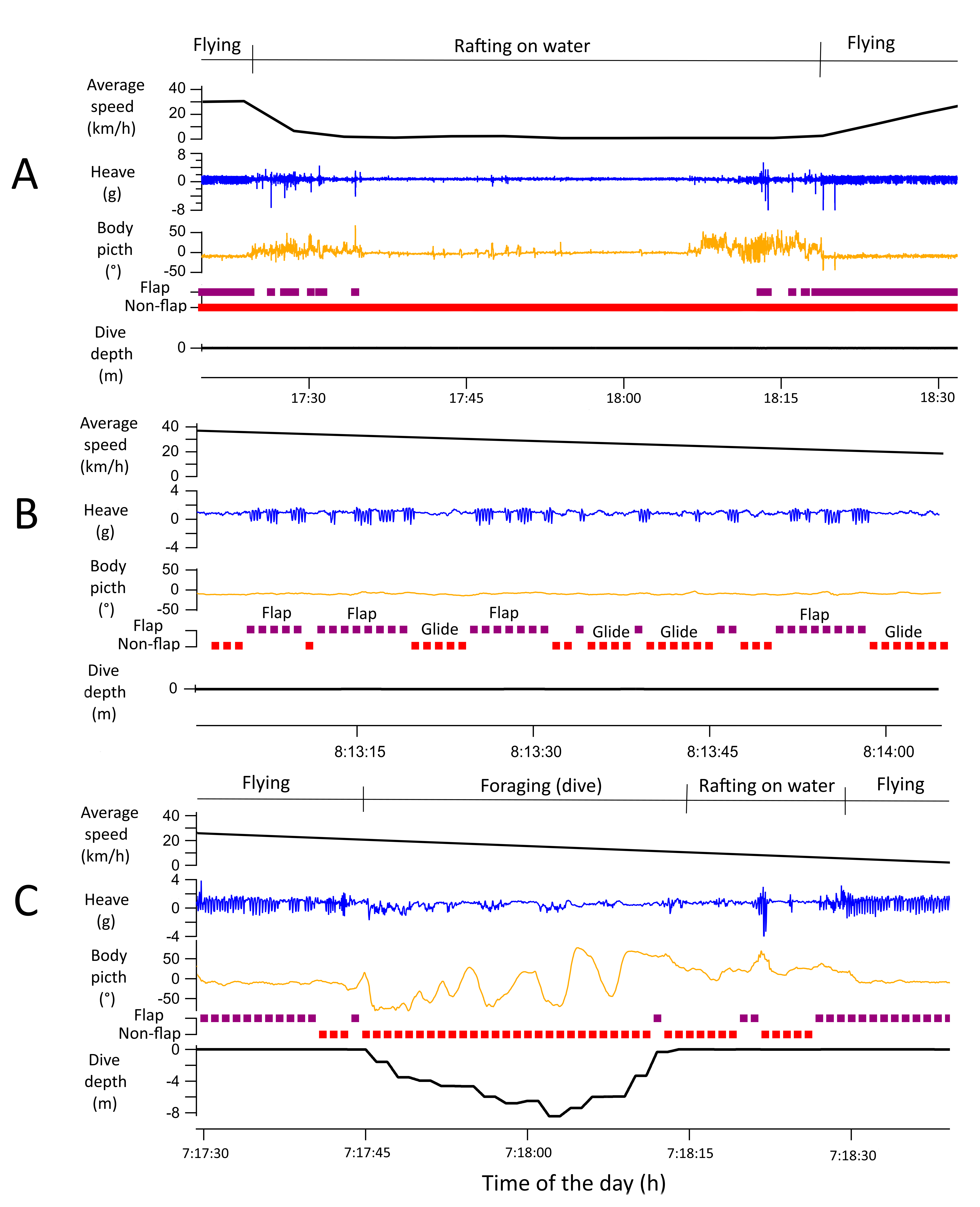

Supplement: Fig S1. At-sea behaviours inferred from tri-axial acceleration [file rsos191511supp1.png]

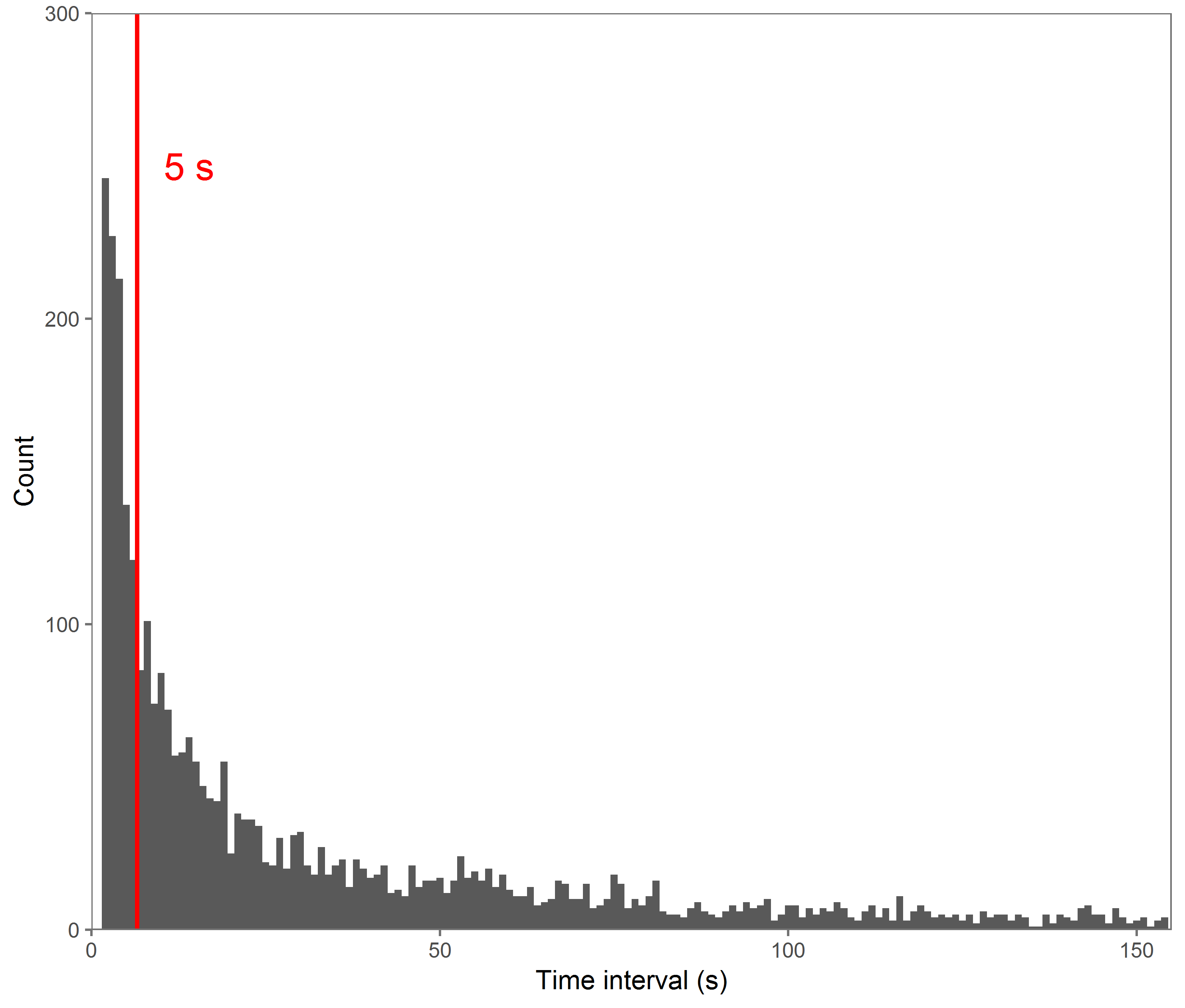

Supplement: Fig S2. Time interval between seconds of foraging [file rsos191511supp2.tif]

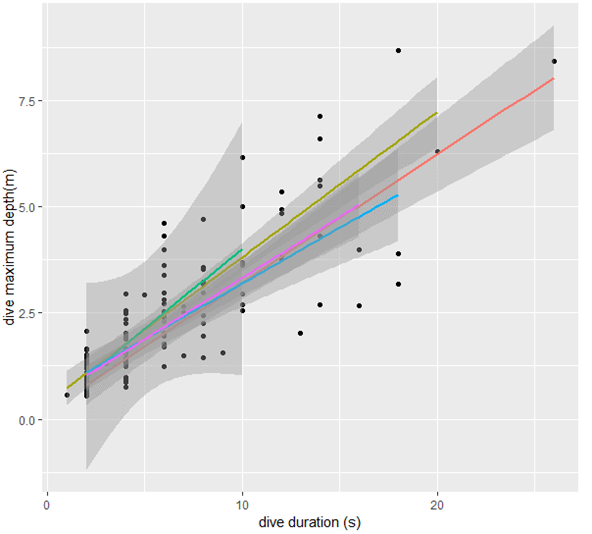

Supplement: Fig S3. Relation between dives duration and depth [file rsos191511supp3.tif]
