# Supplementary material for: Foraging niche overlap during chick-rearing in the sexually dimorphic Westland petrel
Source: R Soc Open Sci. 2020 Nov 25;7(11):191511. doi: 10.1098/rsos.191511 (PMC7735354; doi:10.1098/rsos.191511)
Supplement: Table S1. Oceanographic data [file rsos191511supp4.docx]

Table S 1. Oceanographic data used to model the Westland petrel habitat. DEPTH was downloaded from <https://www.niwa.co.nz/our-science/oceans/bathymetry>, CHLA and ASST from <https://coastwatch.pfeg.noaa.gov/> and the remainders from <http://marine.copernicus.eu/>

| ABBREVIATION, description (unit) | Spatial / temporal resolution | Product |
| --- | --- | --- |
| DEPTH, ocean depth (m) | 0.01° / - | New Zealand Regional Bathymetry 2016 |
| SLOPE, ocean floor slope (°) | 0.01° / - | derivated from DEPTH |
| ASST, SST anomaly (°C) | 0.01° / daily | **MUR SST Analysis Anomaly v04.1** |
| CHLA, chlorophyll a concentration (mg^.^m^-3^) | 0.04 / daily | **Chlorophyll-a, Aqua MODIS, NPP, L3SMI** |
| SST, sea surface temperature (°C) | 0.08° / hourly | Global_Analysis_Forecast_PHY_001_024 |
| SSH, sea surface height (m) | 0.08° / hourly | Global_Analysis_Forecast_PHY_001_024 |
| CURRENT, sea surface water velocity (m^.^s^-1^) | 0.08° / hourly | Global_Analysis_Forecast_PHY_001_024 |
| WAVE, sea surface wave height (m) | 0.08° / 3 h | Global_Analysis_Forecast_Wav_001_027 |
| WIND, wind speed (m^.^s^-1^) | 0.25° / 6 h | Cersat_Glo_Blended_Wind_L4_Rep-V6-Obs |
