# Supplementary material for: Foraging niche overlap during chick-rearing in the sexually dimorphic Westland petrel
Source: R Soc Open Sci. 2020 Nov 25;7(11):191511. doi: 10.1098/rsos.191511 (PMC7735354; doi:10.1098/rsos.191511)
Supplement: Table S2. Models selected [file rsos191511supp5.docx]

Table S 2. Best and most parsimonious models based on AICc explaining the GPS trips parameters, the activity budget and foraging behaviour of Westland petrels rearing chick.

| Model | Response variable | Predictor variable | AICc | Δ AIC | weight | Rho |
| --- | --- | --- | --- | --- | --- | --- |
| GPS trips parameters | | | | | | |
| LMM | Total horizontal distance | year + sex | 530 | 0.0 | 0.9 | - |
| LMM | Trip duration | year + sex | 330 | 0.0 | 0.8 | - |
| LMM | Maximum distance to the colony | year + sex | 405 | 0.0 | 0.9 | - |
| LMM | Average speed | year + sex | 203 | 0.0 | 0.9 | - |
| LMM | Mean VeDBA | Null model | 127 | 0.0 | 0.9 | - |
| Activity budget | | | | | | |
| GAMM | % foraging | s(hour) + year + sex | 349 | 0.0 | 0.3 | -0.005 |
| GAMM | % rafting | s(hour) + year + sex | 15387 | 0.0 | 0.3 | 0.48 |
| GAMM | % flapping | s(hour) + year + sex | 15259 | 0.0 | 0.2 | 0.58 |
| GAMM | % gliding | s(hour) + year + sex | 13249 | 0.0 | 0.2 | 0.61 |
| Foraging behaviour | | | | | | |
| LMM | Foraging rate (time) | Null model | 120 | 0.0 | 0.4 | - |
| LMM | Foraging rate (distance) | Null model | -52 | 0.0 | 0.9 | - |
| GAMM | Dive depth | s(hour) + year + sex | 2420 | 0.0 | 0.2 | 0.17 |
| GAMM | Log(% time foraging)_males_ | s(SST) + s(ASST) + s(WIND) + s(WAVE) + s(CURRENT) + s(DEPTH) + s(SLOPE) | 1766 | 0.7 | 0.3 | 0.13 |
| GAMM | Log(% time foraging)_females_ | s(SST) + s(ASST) + s(DEPTH) + s(SLOPE) | 1715 | 0.05 | 0.2 | 0.14 |
